# Yoga improves self-reported cognitive function among cancer survivors: results from the STAYFit trial

**DOI:** 10.3389/fcogn.2024.1334727

**Published:** 2024-05-10

**Authors:** Neha P. Gothe, Emily Erlenbach, Elizabeth A. Salerno

**Affiliations:** ^1^Bouvé College of Health Sciences, Northeastern University, Boston, MA, United States; ^2^College of Applied Health Sciences, University of Illinois Urbana-Champaign, Urbana, IL, United States; ^3^Division of Public Health Sciences, Department of Surgery, Washington University School of Medicine in St. Louis, St. Louis, MO, United States; ^4^Alvin J. Siteman Cancer Center, Washington University School of Medicine in St. Louis, St. Louis, MO, United States

**Keywords:** *Hatha* yoga, CRCD, cognition, cancer survivorship, physical activity, RCT, chemo brain

## Abstract

**Introduction:**

Various physical activity-based interventions have been tested to determine their efficacy in improving cancer related cognitive decline (CRCD), however the role of mind-body practices such as yoga remains to be explored. In this manuscript we present preliminary effects of yoga vs. aerobic and stretching-toning modalities of exercise on CRCD among adult cancer survivors.

**Methods:**

Participants (*N* = 78) were randomized to one of the three exercise groups for a duration of 12-weeks and engaged in ≥150 min per week of supervised group exercises. At baseline and following the 12-week interventions, participants completed the Functional Assessment of Cancer Therapy–Cognitive Function.

**Results:**

Results demonstrated a significant group^*^time interaction for FACT-Cog perceived cognitive abilities subscale, with participants in the yoga group demonstrating a significant increase as compared to the aerobic and stretching-toning groups. The FACT-Cog total score showed a significant time effect with all groups demonstrating a significant increase at follow-up. Other subscales did not show any significant improvements.

**Discussion:**

These findings provide promising evidence for the effects of yoga on self-reported cognitive function in cancer survivors. Notably, 12-weeks of yoga showed an increase in the perceived cognitive abilities and demonstrated a clinically meaningful increase in total cognitive function as measured by the FACT-Cog, suggesting that this exercise modality has the potential to impact this important health outcome during cancer survivorship.

**Trial registration:**

ClinicalTrials.gov, identifier: NCT03650322.

## Introduction

Cancer is the second leading cause of death globally, accounting for nearly 10 million deaths in 2020 (Ferlay et al., 2021; Sung et al., 2021). Among other symptoms and side effects of cancer treatment and recovery, a lesser known and understood symptom is cancer-related cognitive dysfunction, also referred to as “chemo brain” or cancer-related cognitive decline (CRCD). Patients often define it as a decrease in mental sharpness, mental cloudiness, and being unable to remember certain things, concentrate, finish tasks, or learn new skills (Brant and Stringer, 2015).

Various neuropsychological interventions (Cheng et al., 2022) as well as physical activity-based interventions (Zimmer et al., 2016) have been implemented and tested to determine their efficacy in improving CRCD and overall health and quality of life among cancer survivors. Across several meta-analyses, yoga has been shown to improve symptoms of fatigue, distress, and overall quality of life among cancer survivors (Buffart et al., 2012; Cramer et al., 2012; Harder et al., 2012; Sadja and Mills, 2013). While a review of exercise interventions on CRCD noted that “Asian-influenced movement programs (e.g., yoga)” appear to show preliminary positive effects (Zimmer et al., 2016), another recent systematic review of yoga-based interventions on CRCD (Baydoun et al., 2020) ultimately concluded that there is insufficient evidence for this activity modality and pointed to the need for rigorous trials, expanding populations to beyond breast cancer, and determining the superiority of yoga to more established treatment options or therapies with encouraging evidence for CRCD.

Yoga is an increasingly popular form of complementary and integrative health practice (Clarke et al., 2018) that involves physical movement, breathwork and meditation. A mindful movement practice, yoga enables the practitioner to move slowly and safely into physical postures while concentrating on relaxing the body, breathing fully, and developing awareness of bodily sensations and passing thoughts. The active attentional component of yoga may incur cognitive benefits over and above the habitual bodily movements involved in traditional forms of exercise such as walking or strength training. In addition, breathing and meditation exercises are practiced to calm and focus the mind and develop greater self-awareness. This focused effort and attentional practice of yoga seems to mimic the conventionally assessed cognitive functions including attention, memory, and higher order executive functions. Preliminary studies underscore the role of stress regulation and improved neurocognitive health as mechanisms underlying the yoga-cognition relationship (Voss et al., 2023). The mindful movement practice as well as the potential mechanisms, especially in the context of stress regulation clearly have the potential to be an antidote to the typical cognitive complaints expressed by cancer patients and survivors. In the general population, the effects of yoga on cognitive and brain health are less equivocal, with a recent meta-analysis suggesting that yoga interventions may maintain or improve cognitive function among young and older adults (Gothe and McAuley, 2015).

We have previously reported the feasibility outcomes of this pilot trial where the primary purpose was to determine feasibility and acceptability of yoga and physical activity recommendations for cancer survivors (Gothe and Erlenbach, 2022). Herein we report the secondary data evaluating changes in self-reported symptoms of CRCD in long term cancer survivors following the 12-week supervised *Hatha* yoga intervention, compared with an aerobic walking group and a stretching and toning group. We included the aerobic exercise and stretching and toning conditions as these have been widely used and are among the prescribed exercise guidelines for cancer survivorship (Campbell et al., 2019, 2020). We hypothesized, based on previous literature (Zimmer et al., 2016), that participants across the three exercise conditions would show improvement in self-reported cognitive function following the 12-week exercise intervention. Our exploratory aim was to determine whether participants in the *Hatha* yoga intervention would demonstrate greater improvements in comparison to the aerobic walking and stretching and toning groups.

## Methods

### Recruitment and eligibility procedures

Study design, participant eligibility criteria, and recruitment information have been previously published elsewhere (Gothe et al., 2020). In brief, the primary aim of STAYFit was to test the efficacy of a 12-week Hatha yoga program to improve cognitive function among cancer survivors compared with aerobic walking and stretching-toning active control groups. Low-active cancer survivors who participated in ≤ 2 days per week of structured exercise and were between the ages of 30 and 70 years were eligible. All cancer survivors (except brain) who had completed all cancer-related surgeries, radiation, or chemotherapy sessions at least 1 month prior to study enrollment were invited to participate. No eligibility restrictions were placed on the stage of individuals' cancer diagnosis, or any self-reported cancer related cognitive complaints. Finally, all participants were required to complete the Physical Activity Readiness Questionnaire (PAR-Q) (Thomas et al., 1992) and obtain approval from their personal physician, if deemed high-risk on the PAR-Q. All enrolled participants were randomized into one of the three study arms: Hatha yoga, aerobic walking, or stretching-toning. Recruitment took place in three cohorts and was stratified by age, sex and years since cancer diagnosis.

### Exercise interventions

An in-depth description of the three intervention arms can be found in the published protocol and feasibility manuscripts (Gothe et al., 2020; Gothe and Erlenbach, 2022). Briefly, all exercise groups met for ≥150 min per week. The yoga group met twice weekly for 90 min and practiced *Hatha* yoga, which focused on physical poses, breathing, and meditation and were led by a certified RYT-200 yoga instructor. The frequency and duration of yoga classes was intentionally set to allow the yoga teachers to effectively instruct and demonstrate yoga movements, modifications on and off the floor and with/without yoga blocks, given the novice participant pool. The stretching-toning control arm met three times a week for 60 min and participated in exercises targeting all major muscle groups and worked on balance, toning, and flexibility, with a greater focus on muscle strengthening compared with the yoga group. The aerobic group met three times a week for 60 min and engaged in treadmill walking which increased in intensity and duration over the 12-week period and was individualized to each participant's estimated aerobic fitness level. The stretching and toning group as well as aerobic exercise groups were led by graduate and undergraduate research assistants trained in the instruction and delivery of these exercise modalities as per the American College for Sports Medicine guidelines (Liguori et al., 2022). Attendance was recorded by the exercise instructors and was determined as number of sessions attended/total number of sessions conducted for the 12-week intervention. The stretching and toning group served as an attention-control to standardize social contact with research staff and other participants. This practice allowed for comparisons to a control group with similar resources and attention, which is becoming standard practice for interventions to improve health and wellbeing during cancer survivorship.

The study was approved by the University of Illinois at Urbana Champaign's Institutional Review Board (IRB# 18922). All participants signed a written informed consent document before completing any study assessments. Exercise sessions for the three exercise arms were held on the university campus (except for the latter half of the third cohort, which exercised in their homes due to COVID) in a group-exercise format. All in-person sessions were conducted in exercise studios equipped with necessary exercise equipment.

## Measures

### Demographics and cancer history

Upon meeting the study inclusion criteria, participants completed a demographics survey to document their age, biological sex, race, and education level. Participants were also asked to self-report their cancer history, specifically age at first diagnosis and type of cancer.

### Functional assessment of cancer therapy—Cognitive function

The Functional Assessment of Cancer Therapy–Cognitive Function (FACT-Cog) is a subjective neuropsychological instrument designed to evaluate the effects of cancer patients' perceived cognitive deterioration in their health-related quality of life (Wagner et al., 2009). The Fact-Cog is a valid and reliable questionnaire with excellent internal consistency (Cronbach's alphas for total score and sub-domains > 0.81) (Hajj et al., 2022). This 37-item scale assesses perceived cognitive impairment (20 items, score range 0–80), perceived cognitive abilities (nine items, score range 0–36), quality of life (four items, score range 0–16) and comments from others over the past 7 days (four items, score range 0–16). A total score summing the subscales was also calculated with a possible range from 0 to 148 points. For all subscales and totals, a higher score represents better cognitive functioning or quality of life. Studies have also tried to determine the minimal clinically important difference for the scale which range from 6.9 to 10.6 points (Cheung et al., 2014).

### Data analysis

We used descriptive statistics to summarize the data by group and across the two timepoints. A repeated measures time x group analysis of variance was conducted to determine the main effects of time, group and the interaction between time and group for the FACT-Cog subscales and total score. Attendance was used as covariates across all analyses. A Bonferroni test was applied to test and adjust for multiple comparisons and significance was set at *p* ≤ 0.05. Given the pilot feasibility nature of the study, we examined data for those participants who completed baseline and follow-up surveys and did not apply any corrections or imputations for missing data. Since a subset of the participants transitioned to home-based programming during COVID (Gothe and Erlenbach, 2022), we also analyzed the data with and without this cohort (*n* = 15). There were no significant differences in the results, and we therefore report analyses conducted with all *N* = 78 participants in the trial. SPSS v.28.0 was used for all analyses (IBM Corp., Armonk, NY).

## Results

### Participant characteristics

**Figure 1:**
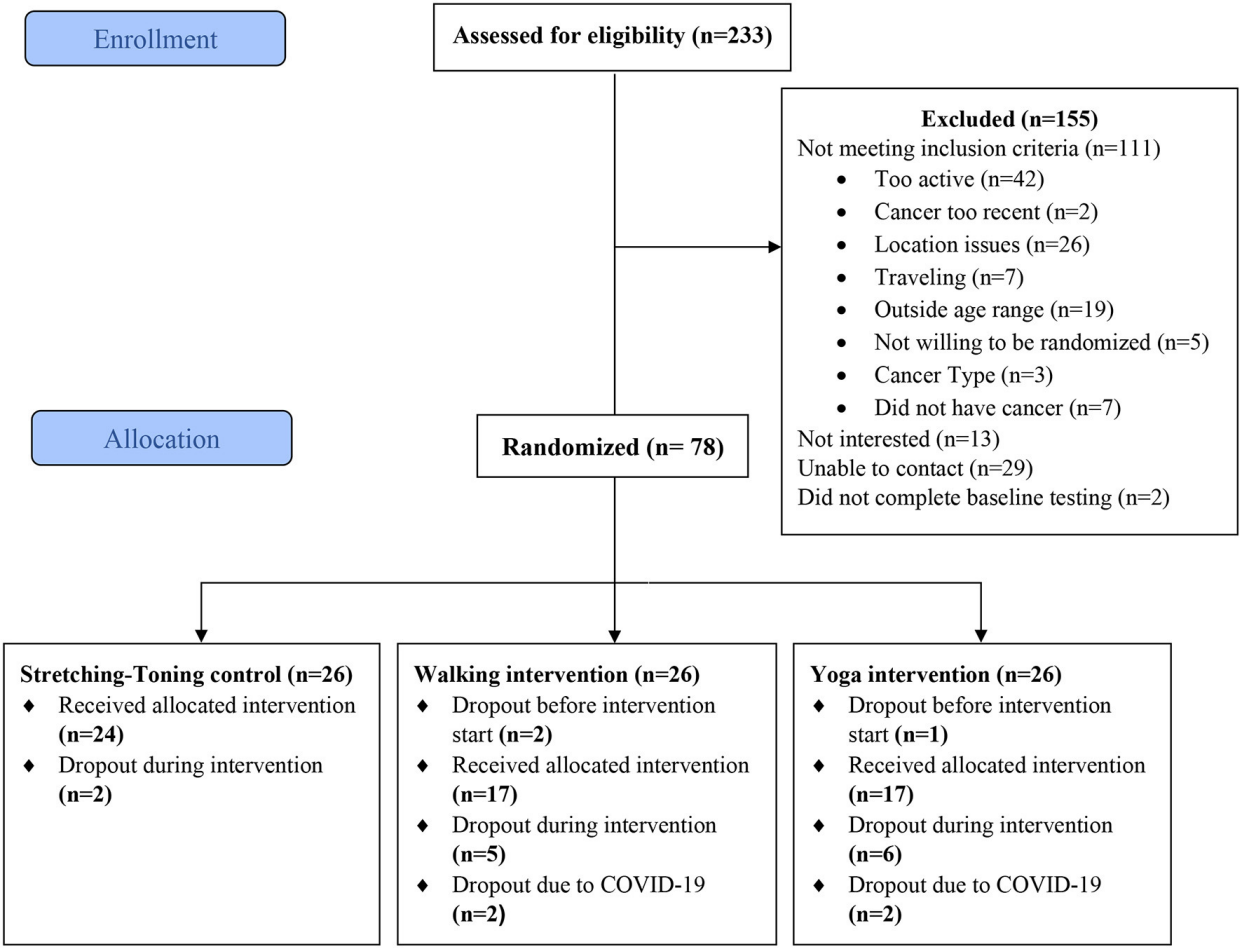
CONSORT for the STAYFit trial. Reproduced from Gothe and Erlenbach (2022).

A detailed study CONSORT is presented in Figure 1. A total of 78 participants (mean age = 55.60, median = 58, age range = 33–69 years) were randomized into one of the three exercise interventions for this trial. A majority of the sample was female (86%) and Caucasian (91%). In terms of cancer characteristics, breast cancer was the most commonly reported (60%) and the average time since diagnosis was 8.32 years (median = 6.2 years, range = 0.5–35.75 years). A detailed breakdown of the participant characteristics is presented in Table 1. The average attendance was 89.53% for the walking group, 76.58% for the stretching and toning group and 77.77% for the yoga group. There was a significant difference in attendance for the walking group as compared to the other two conditions.

**Table 1:** Demographic characteristics of the *N* = 78 cancer survivors in the STAYFit trial.

	**Walking (*n* = 26)**	**Yoga (*n* = 26)**	**Stretching-toning (*n* = 26)**
Age (mean, SD)	55.92 (9.20)	55.00 (9.57)	55.88 (10.70)
**Sex (** ***n*** **, %)**			
Females	23 (88.50)	22 (84.60)	22 (84.60)
Males	3 (11.50)	4 (15.40)	4 (15.40)
**Race (** ***n*** **, %)**			
African American	2 (7.70)	1 (3.80)	2 (7.70)
More than one race	–	1 (3.80)	1 (3.80)
Caucasian	24 (92.30)	24 (92.3)	23.80 (88.50)
**Education (** ***n*** **, %)**			
< College degree	8 (30.80)	8 (30.80)	9 (34.60)
≥College degree	18 (69.20)	18 (69.20)	17 (65.40)
**Cancer type (** ***n*** **, %)**			
Breast	15^*^ (55.55)	18 (69.20)	14 (53.80)
Cervical	–	1 (3.80)	1 (3.80)
Colorectal	1 (3.70)	–	–
Endometrial/uterine	–	1 (3.80)	–
Leukemia	–	–	2 (7.70)
Liver/bile duct	1 (3.70)	–	–
Lung	2 (7.41)	–	–
Lymphoma	2 (7.41)	–	1 (3.80)
Multiple myeloma	–	1 (3.80)	–
Ovarian	3^*^ (11.11)	–	–
Prostate	1 (3.70)	2 (7.70)	–
Sarcoma	–	–	1 (3.80)
Skin	1 (3.70)	2 (7.70)	1 (3.80)
Thyroid	1 (3.70)	1 (3.80)	–
**Stage of cancer diagnosis (** ***n*** **, %)**			
0	1 (3.38)	1 (3.85)	–
1	8 (30.77)	11 (42.31)	8 (30.77)
2	8 (30.77)	8 (30.77)	8 (30.77)
3	4 (15.38)	2 (7.69)	2 (7.69)
4	2 (7.69)	1 (3.85)	2 (7.69)
Don't know	3 (11.54)	3 (11.54)	6 (23.08)
**Cancer treatments**^+^ **(*****n*****, %)**			
Surgery	23 (88.46)	26 (100.00)	24 (92.31)
Radiation	18 (69.23)	16 (61.54)	13 (50.00)
Chemotherapy	13 (50.00)	12 (46.15)	13 (50.00)
Years since diagnosis (mean, SD)	7.72 (7.40)	8.29 (8.64)	8.94 (6.92)

^*^One participant had both breast and ovarian diagnoses; percentages are calculated out of *n* = 26 cases.

^+^Majority of participants reported undergoing more than one cancer treatment.

### FACT-Cog

The means and standard deviations on the FACT-Cog subscales by groups and across timepoints are presented in Table 2. For the FACT-Cog subscales of perceived cognitive impairment, comments from others, and impact on quality of life, there were no significant time, group or interaction effects (*p'*s > 0.10). Although not statistically significant, the mean scores across all groups for these subscales did show an increase over time as observed in Table 2.

**Table 2:** Means and standard deviations on the Functional Assessment of Cancer Therapy—Cognitive Function scale.

	**Yoga**		**Walking**		**Stretching-toning**	
	**Baseline**	**12-week**	**Baseline**	**12-week**	**Baseline**	**12-week**
FACT-Cog—Perceived Cognitive Impairments	60.28 (12.16)	66.14 (12.62)	61.81 (15.47)	65.67 (12.06)	61.04 (14.39)	64.87 (11.93)
FACT-Cog—Comments from Others	14.71 (2.59)	15.33 (1.02)	15.29 (1.85)	15.52 (1.44)	15.39 (1.88)	15.52 (1.16)
FACT-Cog—Perceived Cognitive Abilities	26.38 (5.11)	29.86 (5.02)	25.00 (8.00)	26.33 (7.47)	24.91 (7.19)	24.96 (6.82)
FACT-Cog—Quality of Life	12.52 (3.16)	12.71 (3.33)	12.52 (3.97)	13.19 (3.64)	13.30 (3.41)	13.57 (3.66)
FACT-Cog—Total score	113.90 (15.99)	124.04 (16.41)	114.62 (26.23)	120.71 (20.70)	114.65 (17.46)	118.91 (14.64)

FACT-Cog, Functional Assessment of Cancer Therapy—Cognitive Function scale.

For the FACT-Cog perceived cognitive abilities subscale, there was a significant time x group interaction effect [*F* = 3.03_(2, 61)_, η^2^ = 0.09, *p* = 0.05]. A *post-hoc* Bonferroni test revealed that participants in the yoga group showed a significant increase in their perceived cognitive abilities from baseline to 12-weeks (Mean difference = 3.55, SE = 1.04, *p* < 0.01) whereas no significant changes were reported within the walking and stretching-toning groups. A closer examination of the physical cognitive abilities subscale in terms of the participants who reported increases, no change and decreases are presented in Table 3. These were calculated as raw score changes (post-intervention—baseline) for each participant who completed the trial. Overall, the percentage of participants in the yoga group who reported an increase was higher compared to those in the aerobic and stretching-toning groups as well as those who reported declines was lower in the yoga group in comparison to the other two.

**Table 3:** Number and percentages of participants that improved, showed no change and declined on the FACT-Cog Perceived Cognitive Abilities subscale.

	**Yoga (*n* = 21)**	**Aerobic (*n* = 21)**	**Stretching-toning (*n* = 23)**
**FACT-Cog—Perceived Cognitive Abilities**			
Improved	17 (81%)	13 (62%)	11 (48%)
No change	3 (14%)	2 (10%)	4 (17%)
Declined	1 (5%)	6 (28%)	8 (35%)

Numbers and percentages calculated on the raw Fact-Cog Perceived Cognitive Abilities score (post intervention—baseline).

For the FACT-Cog total score, the main effect of time was marginally significant [*F* = 3.60_(1, 61)_, η^2^ = 0.06, *p* = 0.06] and no significant group^*^time effect was observed. *Post-hoc* pairwise comparisons revealed that all exercise groups, yoga (mean difference = 9.98, SE = 2.36, *p* < 0.01), walking (mean difference = 6.15, SE = 2.33, *p* = 0.01), and stretching-toning (mean difference = 4.35, SE = 2.24, *p* = 0.05) showed a significant increase from baseline to 12-weeks, with the yoga group demonstrating the largest increase.

## Discussion

To our knowledge, this is the first clinical trial to test the preliminary effect of yoga vs. aerobic and stretching-toning modalities of exercise on CRCD among adult cancer survivors. Our results add to the existing literature on exercise interventions to combat CRCD suggesting that yoga, aerobic walking as well as stretching-toning exercises can be useful to reduce self-reported cognitive symptoms, and *Hatha* yoga particularly could improve cancer survivors perceived cognitive abilities.

Assessment of CRCD has varied across the studies in the literature and this diversity has caused considerable heterogeneity in the studies, making comparisons difficult and conclusions subjective (Henneghan et al., 2021). We choose to use the FACT-Cog as it is a well-documented and validated instrument, and one of the few which has a minimal clinically important change value published in the literature (Cheung et al., 2014). In our study, the FACT-Cog total change scores from baseline to 12-weeks were 9.86 for yoga, 6.09 for walking and 4.26 for stretching-toning groups. The change score for yoga was within the clinically meaningful range (Cheung et al., 2014), suggesting that yoga may present cognitive benefits above and beyond other exercise modalities for this specific cancer-related outcome. Indeed, Campbell and colleagues have noted varying, outcome-specific exercise prescriptions in recent guidelines for cancer survivors (Campbell et al., 2019). Consensus from the international multidisciplinary panel that drafted these exercise guidelines highlighted the paucity of evidence for the effects of exercise on cognitive function. Our findings suggest that yoga, aerobic, and stretching and toning exercise, all may incur benefits for cognitive health when delivered in a group exercise format for inactive long term cancer survivors.

A handful of studies have examined the effects of yoga interventions on CRCD. Derry et al. (2015) reported 23% fewer cognitive problems as assessed by the Breast Cancer Prevention Trial Cognitive Problems Scale at a 3-month follow-up among breast cancer patients who had practiced Hatha yoga for 12 weeks when compared with a waitlist control group. Similarly, Janelsins et al. (2016) reported significant improvements (19%) in memory among cancer survivors who participated in a 4-week yoga program compared with those in a standard survivorship program. More recently, in a single group, proof of concept study, an 8-week virtual yoga intervention showed significant improvements on objective cognitive function among 18 breast cancer survivors but no significant improvements on FACT-Cog scores (Neville et al., 2023). While these studies provided preliminary evidence for the efficacy of yoga on CRCD, the lack of an active comparator and study population limited to breast cancer survivors limits the generalizability of the findings to all cancer survivors. Our findings contribute to this nascent but growing literature base by highlighting the benefits of yoga for improving overall cognition, as measured by the FACT-Cog, in cancer survivors of all types within a randomized controlled design. A closer look at the trends in subscales, specifically the perceived cognitive abilities subscale indicated that a majority of the yoga participants, 81% showed an increase and only 1 subject declined when compared to the other two groups (Table 3). The specific group differences that emerged on this subscale are not surprising as several of the subscale items directly map onto yoga-based mindfulness practices and interoception. For example, “able to concentrate,” “able to pay attention,” “able to shift back and forth,” “able to keep track” are often practiced in the context of yoga-based breathing exercises, i.e., *pranayama* and meditation, i.e., *dhyana* which were part of the *Hatha* yoga protocol for this study. It is possible that the participants in the yoga group were able to see the translation of the yoga practices from “on the mat” (in class) to “off the mat” (in their day-to-day) cancer survivorship symptoms and experiences. These nuanced results also highlight the need for alternative yoga protocols with varying focus on breathing and mindfulness to be explored in future, larger clinical trials investigating the exercise-cognition relationship during cancer survivorship.

There are several biopsychosocial mechanisms through which yoga may influence cancer survivors' cognitive function. CRCD is a complex, multifactorial phenomenon; inflammation, psychosocial factors (e.g., anxiety, depression), changes in brain structure and function, host factors (e.g., sociodemographic factors, genetics), and behavior (e.g., diet, exercise) all converge to affect a survivors' cognitive function after cancer (Janelsins et al., 2014; Wefel et al., 2015; Lange et al., 2019). Exercise is well-known for influencing inflammatory pathways, neurotransmitter expression, glucose signaling, and hormone regulation (Erickson et al., 2019). Yoga specifically has been associated with reductions in inflammation (Kiecolt-Glaser et al., 2014), and its mind-body principles may promote mindfulness and reduce anxiety (Maloney et al., 2014), both of which have been associated with cognitive function (Chiesa et al., 2011; Voss et al., 2023). Morphological and functional brain imaging studies show that yoga practice has an effect on brain areas involved with interoception, posture, motivation, and higher order executive functions such as the hippocampus, amygdala, insula, prefrontal and frontal cortices (Gothe et al., 2019; Van Aalst et al., 2020). This is particularly interesting as a meta-analysis of chemo-brain was linked to deficits in the same regions of interest, the frontal attention network (Bernstein et al., 2021). Patients treated with chemotherapy demonstrate hypo-activity, i.e., a dysfunction in mobilizing attentional resources in the frontal-parietal cortices during cognitive tasks. Therefore, yoga may be a more potent therapy to address CRCD given the focus on sustained attention and mindfulness that is embedded in its practice as compared to other forms of exercise tested in this trial. Future studies should include imaging and biomarkers to better understand the effects of yoga on self-reported as well as objectively assessed CRCD.

A notable characteristic of our sample was the long-term survivorship, with patients reporting on average 8.32 years since their diagnosis. This is longer than many previous studies (Campbell et al., 2020) and proves the persistence of cognitive complaints among cancer survivors years after diagnosis and treatment. Our results demonstrate that lifestyle behaviors such as exercise have the potential to impact CRCD long after diagnosis. To improve adoption and adherence, novel approaches to randomized controlled designs, such as preference-based group assignments, should be explored. In our trial, we noted higher attendance for the aerobic exercise group where participants were in smaller groups and had more one-on-one interactions with the research staff as they walked on a treadmill. Given the evidence for group cohesion and social connectedness (Gothe et al., 2019), identifying the social dynamics that support adherence after cancer would help researchers better design exercise programs that could lead to sustained adherence and thereby result in improved CRCD and health outcomes.

This study has several strengths. To our knowledge, this is the first randomized controlled trial to explore the effects of yoga on cognitive function in multiple comparison groups during cancer survivorship. Attendance in all three groups was excellent (>75%) with no adverse events. This study enrolled survivors of various cancer types in an attempt to increase generalizability of our results to all of cancer survivorship, a noted gap in the literature. Despite these strengths, our findings should be interpreted within the context of various limitations. The current sample was predominantly female and White with a history of breast cancer. Each of the three intervention groups were comprised of fewer than 30 participants, further limiting our ability to explore differential effects by important cancer-related constructs (i.e., cancer stage and type, time since diagnosis). For future studies, it will be important to understand if and how the effects of yoga on CRCD differ within these clinical groups to inform the design of personalized behavioral interventions. To this extent, a true control, such as a waitlist group or health education group (i.e., no exercise condition), could also offer meaningful insights. Similarly, although we met our target sample size, our study was hampered by the pandemic and a subset of the participants ended up having a unique experience with the intervention with a combination of in-person and virtual instruction. Future studies, with larger sample sizes, could also adopt mobile health (mHealth) approaches to identify the most effective mode of exercise intervention delivery for cognitive benefits among cancer survivors. Finally, cognitive function was self-reported and therefore may have captured other domains (i.e., mood, fatigue) resulting in misclassification (Wefel et al., 2011). While self-reported cognitive function is crucial for understanding and legitimizing survivors' lived experience with CRCD (Henneghan et al., 2021), there is noted discordance in the literature between objective and self-reported measures of CRCD (Horowitz et al., 2018). Self-reported cognitive symptoms could be misattributed to one's cancer experience, other health conditions, or normative aging. Future studies should include both self-report and standardized objective measures of cognitive function (Campbell et al., 2019) to fully characterize the yoga-cognition relationship.

In conclusion, these findings provide promising support for the effects of yoga on self-reported cognitive function in cancer survivors. Notably, 12 weeks of group-based, hybrid *Hatha* yoga demonstrated a clinically meaningful increase in patient reported cognitive function, suggesting that this exercise modality may be preferable for this important health outcome during survivorship. As individuals continue to live years beyond a diagnosis of cancer, understanding the continued impact of cancer on CRCD and testing lifestyle interventions such as exercise and yoga to maximize cognitive health and overall quality of life should remain a high priority.

## Data availability statement

The data that support the findings of this study are available from the corresponding author, [NG], upon reasonable request.

## Ethics statement

The study involving humans were approved by Office for Protection of Research Subjects, M/C 685 1901 S. First St, Suite A Champaign, IL 61820 217-333-2670, irb@illinois.edu. The study was conducted in accordance with the local legislation and institutional requirements. The participants provided their written informed consent to participate in this study.

## Author contributions

NG: Conceptualization, Data curation, Formal analysis, Funding acquisition, Investigation, Methodology, Project administration, Resources, Supervision, Writing—original draft, Writing—review & editing. EE: Data curation, Investigation, Methodology, Project administration, Supervision, Writing—review & editing. ES: Writing—original draft, Writing—review & editing.
